# Role of Arctiin in Fibrosis and Apoptosis in Experimentally Induced Hepatocellular Carcinoma in Rats

**DOI:** 10.7759/cureus.51997

**Published:** 2024-01-10

**Authors:** Shahad A Alshehri, Wasayf A Almarwani, Ajwan Z Albalawi, Shekha M Al-atwi, Khulud K Aljohani, Amjad A Alanazi, Mohamed A Ebrahim, Hanan M Hassan, Mohammed M Al-Gayyar

**Affiliations:** 1 Pharmacy, University of Tabuk Faculty of Pharmacy, Tabuk, SAU; 2 Pharmacy, King Salman Armed Forced Hospital, Tabuk, SAU; 3 Medical Oncology, Oncology Center, Mansoura University, Mansoura, EGY; 4 Pharmacology and Biochemistry, Delta University for Science and Technology, Gamasa, EGY; 5 Pharmaceutical Chemistry, University of Tabuk Faculty of Pharmacy, Tabuk, SAU; 6 Biochemistry, Mansoura University Faculty of Pharmacy, Mansoura, EGY

**Keywords:** protein kinase c (pkc), mothers against decapentaplegic homolog 4 (smad4), hypoxia-induced factor-1α (hif-1α), hepatocellular carcinoma (hcc), extracellular signal-regulated kinase (erk), β-catenin

## Abstract

Background and objectives

Hepatocellular carcinoma (HCC) is a highly aggressive malignant tumor with a poor prognosis. It is currently the second most common cause of cancer-related mortality. Arctiin, a compound found in plants commonly used as a vegetable in Asian countries and as an ingredient in traditional European dishes, possesses various properties, including anti-proliferative, anti-senescence, anti-oxidative, anti-tumor, toxic, anti-adipogenic, and anti-bacterial effects. Our study aims to investigate the potential antitumor activity of arctiin against HCC in rats by inhibiting cell fibrosis and apoptosis.

Methods

Rats were induced with HCC by administering thioacetamide. Arctiin was orally administered to some rats twice a week for 16 weeks at a dose of 30 mg/kg. The liver impairment was evaluated by measuring serum α-fetoprotein (AFP) and examining liver sections stained with Masson trichrome or anti-hypoxia-induced factor-1α (HIF-1α) antibodies. The hepatic expression of messenger RNA and protein levels of HIF-1α, protein kinase C (PKC), extracellular signal-regulated kinase (ERK), β-catenin, and mothers against decapentaplegic homolog 4 (SMAD4) were analyzed.

Results

Our study demonstrated that arctiin can potentially increase the survival rate of rats. This is achieved through a reduction in serum AFP levels and hepatic nodules. We also observed that arctiin has the ability to inhibit the formation of fibrotic tissues and necrotic nodules in HCC rats. Additionally, arctiin can significantly decrease the expression of HIF-1α, PKC, ERK, β-catenin, and SMAD4.

Conclusion

Arctiin has demonstrated potential anti-tumor properties that could ameliorate HCC. Studies have shown that it may increase survival rates and reduce the number of tumors and AFP levels. Arctiin works by inhibiting HCC-induced hypoxia, thus blocking the expression of HIF-1α. It also helps to slow down tumor fibrosis by decreasing the expression of β-catenin and SMAD4. Furthermore, arctiin has been found to downregulate PKC and ERK, reducing hepatic tissue apoptosis.

## Introduction

Hepatocellular carcinoma (HCC) is a highly aggressive type of liver cancer and is currently the sixth most common cancer worldwide, causing approximately 600,000 deaths and 1,000,000 new cases each year. Unfortunately, HCC has limited treatment options and a high mortality rate of less than 12% [1]. Despite numerous efforts to prevent, detect, diagnose, and treat HCC, the death rate continues to rise. HCC is known to spread to other parts of the body and has a high chance of recurrence even after treatment. Moreover, the lack of targeted molecular therapies available for HCC results in poor prognoses, making it a fatal disorder [2].

When liver cancer is not treatable through surgery or has spread to other organs, initial treatment involves prescribing kinase inhibitors such as sorafenib or lenvatinib. In cases of advanced liver cancer, regorafenib and cabozantinib are used. Currently, hepatic resection or transplantation is considered the only curative therapy for HCC [3]. However, even with surgery, around 30% of HCC patients undergo hepatectomy as they receive a diagnosis at an advanced tumor stage [4]. Radiofrequency ablation is a treatment used for patients diagnosed with early stage HCC [5]. Unfortunately, several factors such as a history of cirrhosis, poverty, and limited medical resources lead to a poor prognosis for patients with HCC [6]. Moreover, it has a high angioinvasive capacity due to portal vein obstruction. Therefore, there is an urgent need to identify, develop, and improve the overall novel therapeutic drugs for the treatment of HCC [7].

Lignans are a group of natural chemical compounds that are abundantly present in various seeds, beans, fruits, and vegetables. They are formed by binding phenylpropane structures together and are known to play a crucial role in many biological processes, including hormone metabolism, cell differentiation, transformation, and proliferation [8]. Arctiin is one of the well-known lignans that can be found in the Arctium lappa L plant, which is a common ingredient in traditional European dishes and is used as a vegetable in Asian countries [9]. Numerous scientific studies have demonstrated that arctiin possesses a range of health benefits, including anti-proliferative, anti-senescence, anti-oxidative, anti-tumor, toxic, anti-adipogenic, and anti-bacterial properties [10].

Given these promising properties, researchers are currently investigating the potential of arctiin as a treatment for cancer. Specifically, the aim of the study is to examine the in vivo anti-cancer effects of arctiin in rats with HCC. Additionally, the study aims to investigate the potential antitumor activity of arctiin against HCC in rats by investigating its effects on the expression of β-catenin, extracellular signal-regulated kinase (ERK), hypoxia-induced factor-1α (HIF-1α), mothers against decapentaplegic homolog 4 (SMAD4), and protein kinase C (PKC).

## Materials and methods

Animals

All animal experiments were conducted following the appropriate guidelines and were approved by the Faculty of Pharmacy, Delta University for Science and Technology Research Ethics Committee (number FPDU8/2023), Gamasa, Egypt. Forty male Sprague Dawley rats, aged 8-10 weeks and weighing 150-200 g, were used for the study. The rats were housed in stainless-steel cages under standard conditions, with a temperature of 25±1°C and a 12-hour light and dark cycle. The rats were divided into the following four groups, with 10 rats in each group:

1. *Control group*: rats were injected with normal saline for 16 weeks daily.

2. *Arctiin-treated control group*: rats were given 30 mg/kg arctiin (Sigma Aldrich Chemicals Co, St. Louis, MO) via oral gavage daily for 16 weeks.

3. *HCC group*: rats were given 200 mg/kg of thioacetamide in normal saline via intraperitoneal injection twice weekly for 16 weeks.

4. *Arctiin-treated HCC group*: rats were given 200 mg/kg thioacetamide in normal saline intraperitoneally twice weekly and 30 mg/kg arctiin orally daily for 16 weeks.

Sample collection

Rats were anesthetized with intraperitoneal thiopental sodium (40 mg/kg). Blood samples were collected from the retro-orbital plexus and then centrifuged at 3000 rpm for 5 minutes. The serum samples were stored at -80°C. To investigate its morphological features, a portion of the hepatic loop was separated, sliced, and kept in a 10% buffered formalin solution. Another part of the hepatic loop was homogenized in a 10-fold volume of phosphate buffer at pH 7.4 and stored at -80°C.

Histopathological examination

Hepatic slices, which were kept in 10% formalin, were embedded in paraffin blocks and then cut into 5-μm-thickness sections. These sections were stained with Masson trichrome to investigate the structure of hepatic tissues. To ensure impartial examination, the sections were coded anonymously and examined in a masked manner. For immunohistochemistry, the sections were incubated with monoclonal anti-HIF-1α (Sigma-Aldrich) at 4°C. After that, the sections were incubated with horseradish peroxidase-conjugated antibody. Then, 2% of 3,3′-diaminobenzidine in tris-buffer was used as a chromogen. Finally, hematoxylin was applied as a counterstain [11-13].

Enzyme-linked immunosorbent assays (ELISA) determination

Commercially available enzyme-linked immunosorbent assays (ELISA) kits were used for the assessment of α-fetoprotein (AFP), HIF-1α, β-catenin, SMAD4, ERK, and PKC (MyBioSource, Inc., San Diego, CA, USA) according to the instructions of the manufacturer.

Quantitative real-time polymerase chain reaction

The levels of messenger RNA for HIF-1α, β-catenin, SMAD4, ERK, and PKC were measured in rat hepatic lysate using methods previously described by our group [14-16]. GAPDH (glyceraldehyde-3-phosphate dehydrogenase) was used as a reference control. The specific polymerase chain reaction (PCR) primers used for each gene are summarized in Table 1.

**Table 1:** Primer sets used to detect gene expression in rats. ERK, extracellular signal-regulated kinase; GAPDH, glyceraldehyde-3-phosphate dehydrogenase; HIF-1α, hypoxia-induced factor-1α; PKC, protein kinase C; SMAD4, mothers against decapentaplegic homolog 4

Name	Sequence		Reference sequence
GAPDH	Forward	5`-CCATCAACGACCCCTTCATT-3`	NM_017008.4
	Reverse	5`-CACGACATACTCAGCACCAGC-3`	
HIF-1α	Forward	5`-GCAACTAGGAACCCGAACCA-3`	NM_024359.2
	Reverse	5`-TCGACGTTCGGAACTCATCC-3`	
β-catenin	Forward	5`-TCCGTCGCCGGTCCACACCC-3`	NM_031144.3
	Reverse	5`-TCACCAACTGGGACGATATG-3`	
SMAD4	Forward	5`-GGATGAAGTCCTGCACACCA-3`	NM_019275.3
	Reverse	5`-GTTGAAGCACTGCCACCTTG-3`	
ERK	Forward	5`-CCCCTTCGAGCATCAAACCT-3`	M38194.1
	Reverse	5`-TTCTCATGGCGGAATCCGAG-3`	
PKC	Forward	5`-GTAACTTGAACCCTCATCTCCT-3`	X04439.1
	Reverse	5`-CAAGTGTTCTTAGCCGCACG-3`	

Statistical analysis

The study presented its results as mean ± SEM. The distribution of samples in the study was checked for normality using the Kolmogorov-Smirnov test. The survival rate of rats was checked using the Kaplan-Meier procedure. The study used one-way analysis of variance (ANOVA) followed by the Bonferroni post hoc test to determine significant differences among groups. The statistical analysis was conducted using SPSS Version 20 (IBM Corp., Armonk, NY, USA). A p-value of less than 0.05 indicated a statistically significant difference.

## Results

Antitumor activity of arctiin

**Figure 1:**
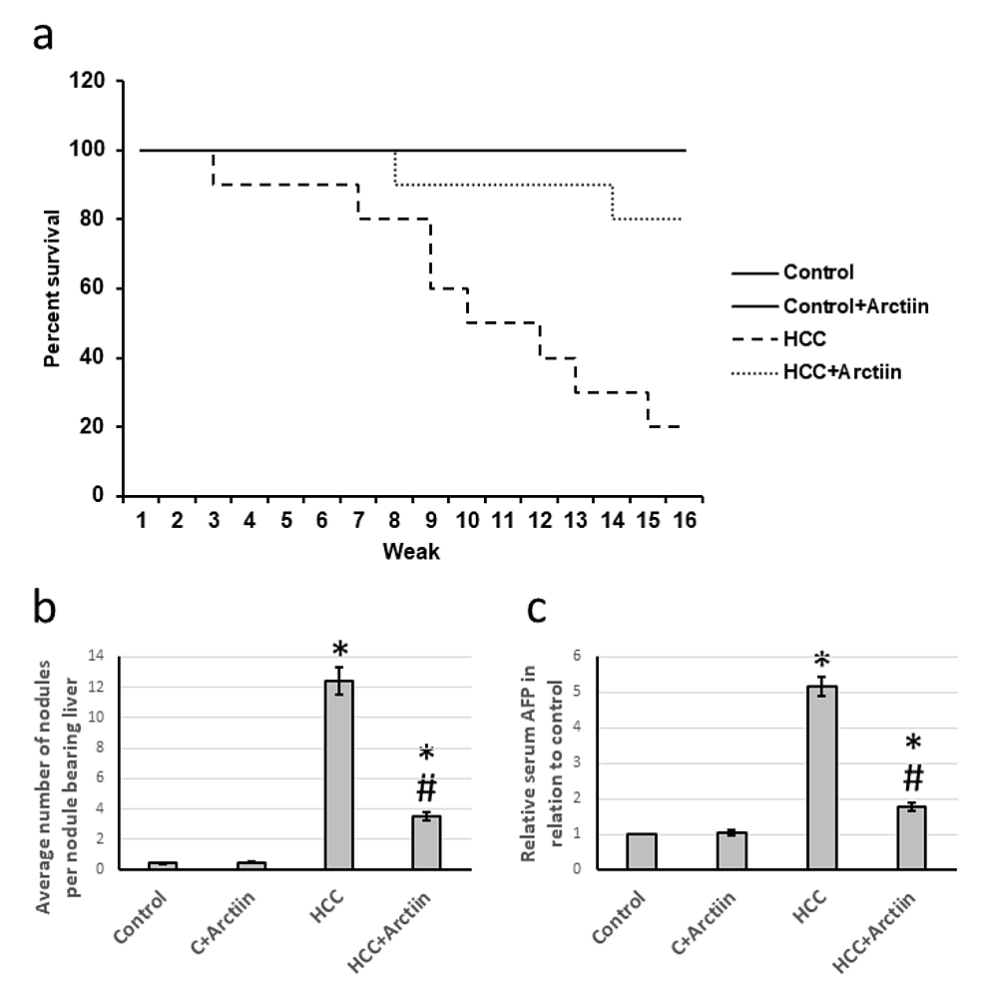
Effect of HCC and 30 mg/kg arctiin on rats’ survival (a), the average number of nodules (b), and serum AFP (c). *Significant difference as compared with the control group at p<0.05. #Significant difference as compared with the HCC group at p<0.05. AFP, alpha-fetoprotein; C, control; HCC, hepatocellular carcinoma

In a study conducted on rats with HCC, the treatment with arctiin resulted in a significant increase in the survival rate. Around 80% of the rats survived, compared to only 20% in the HCC group that did not receive treatment. Moreover, the arctiin-treated group showed a significant reduction in the average number of nodules in the liver. The study also revealed a decrease in serum AFP levels compared to those in the HCC group (Figure 1).

Effect of arctiin and HCC on the structure of hepatic tissue

**Figure 2:**
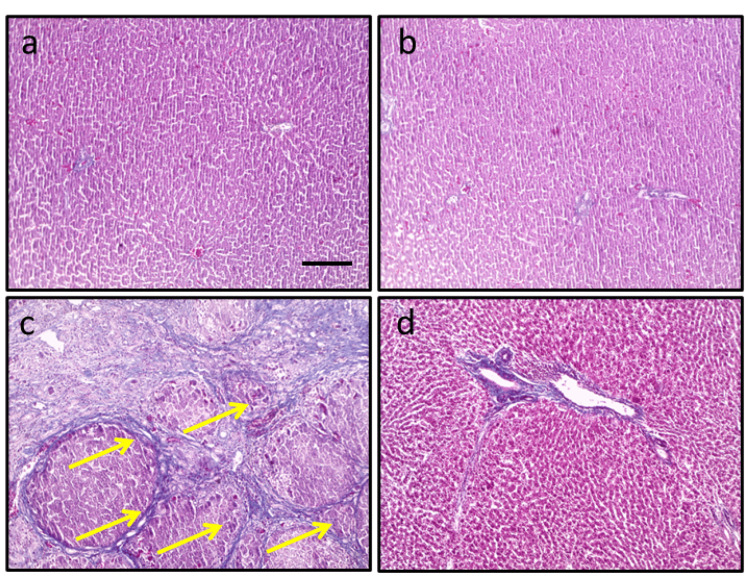
Hepatic sections stained with Masson trichrome in the control group (a), control group treated with arctiin (b), hepatocellular carcinoma group (c), and hepatocellular carcinoma group treated with arctiin (d). Yellow arrows indicated areas of fibrosis. Scale bar represents 100 μm.

In the control group, the microsection stained with Masson trichrome showed normal tissues with no significant changes or abnormalities. However, in the HCC group, an increased area of fibrosis was observed. Conversely, when sections from HCC rats treated with arctiin were examined, there was a marked improvement in hepatic tissue, with a reduction in the fibrosis area. These improvements may include reduced cellular abnormalities or a decrease in the size of cancerous tissues, as shown in Figure 2.

Effect of arctiin and HCC on the expression of HIF-1α

**Figure 3:**
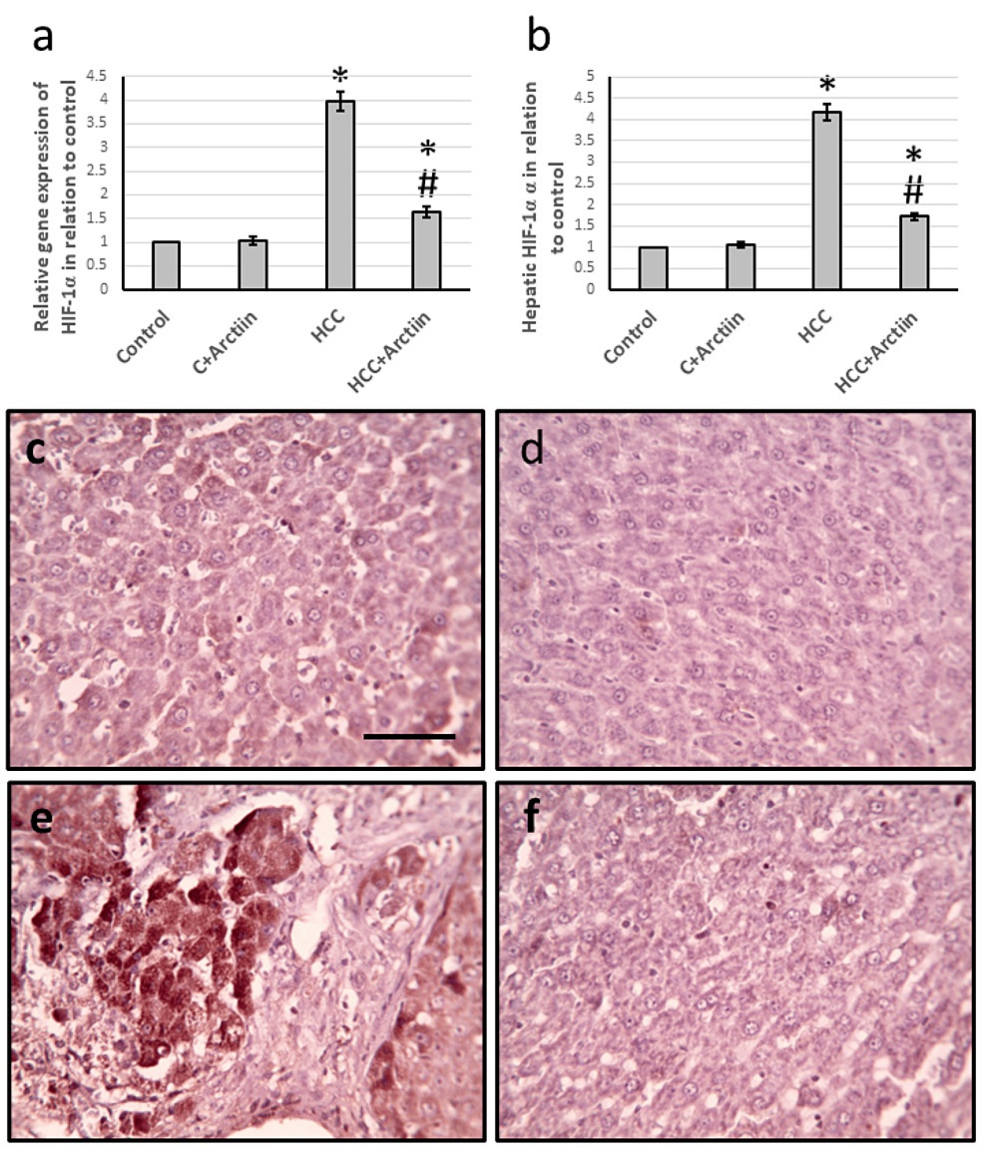
Effect of HCC and 30 mg/kg arctiin on hepatic gene expression of HIF-1α (a) and its hepatic protein level (b). Hepatic sections stained with anti-HIF-1α antibodies in the control group (c), control group treated with arctiin (d), HCC group (e), and HCC group treated with arctiin (f). *Significant difference as compared with the control group at p<0.05. #Significant difference as compared with the HCC group at p<0.05. Scale bar 50 μm. C, control; HCC, hepatocellular carcinoma; HIF-1α, hypoxia induced factor-1α

In experiments conducted on rats with HCC, it was found that the gene expression of HIF-1α increased significantly by 3.98-fold, along with a 4.18-fold increase in the levels of HIF-1α protein in the liver. The liver sections that were stained with anti-HIF-1α antibodies also showed a considerable increase in the stained areas. However, when treated with arctiin, the effects were reversed in the HCC group without affecting the control groups, as shown in Figure 3.

Effect of arctiin and HCC on the expression of β-catenin and SMAD4

**Figure 4:**
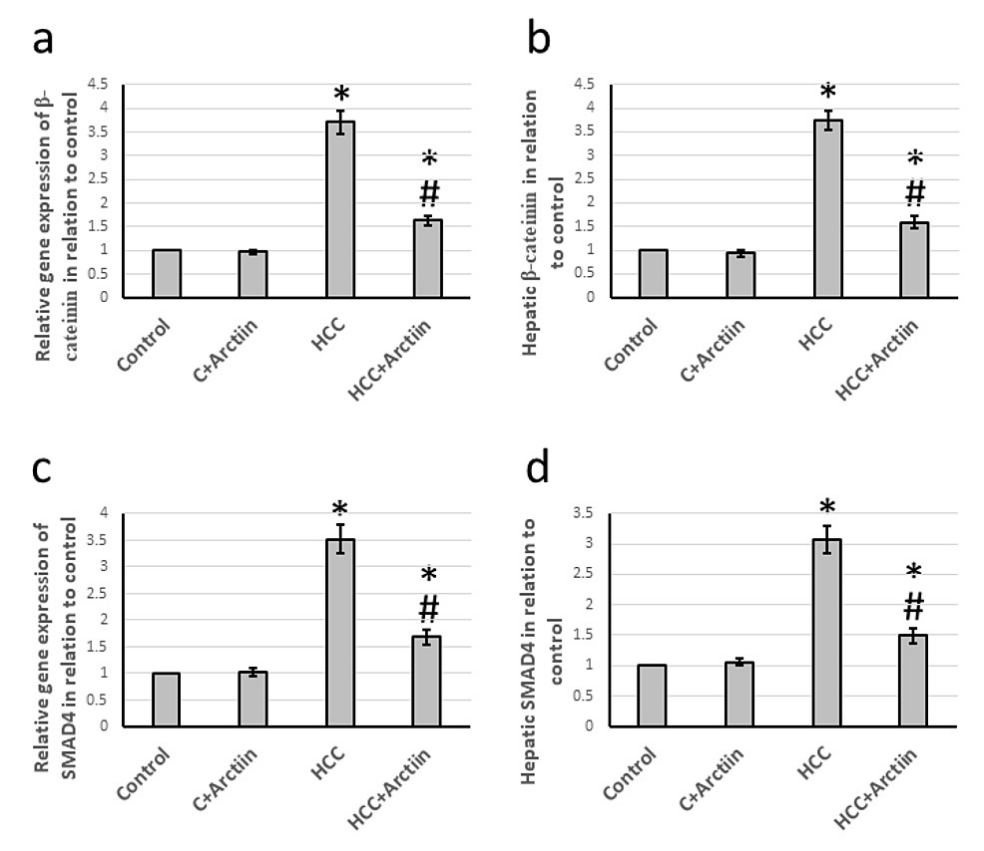
Effect of HCC and 30 mg/kg arctiin on hepatic gene expression of β-catenin (a) and SMAD4 (c), as well as protein expression of β-catenin (b) and SMAD4 (d). *Significant difference as compared with the control group at p<0.05. #Significant difference as compared with the HCC group at p<0.05. C, control; HCC, hepatocellular carcinoma; SMAD4, mothers against decapentaplegic homolog 4

During the examination of liver tissues from rats with HCC, it was found that there was a significant increase in the gene expression of β-catenin and SMAD4 by 3.71- and 3.52-fold, respectively. Moreover, the hepatic tissue levels of catenin and SMAD4 were also elevated by 3.74- and 3.07-fold, respectively, as compared to the control group. However, the administration of arctiin was able to reverse all of these effects in HCC rats without affecting the control rats (Figure 4).

Effect of arctiin and HCC on the expression of PKC and ERK

**Figure 5:**
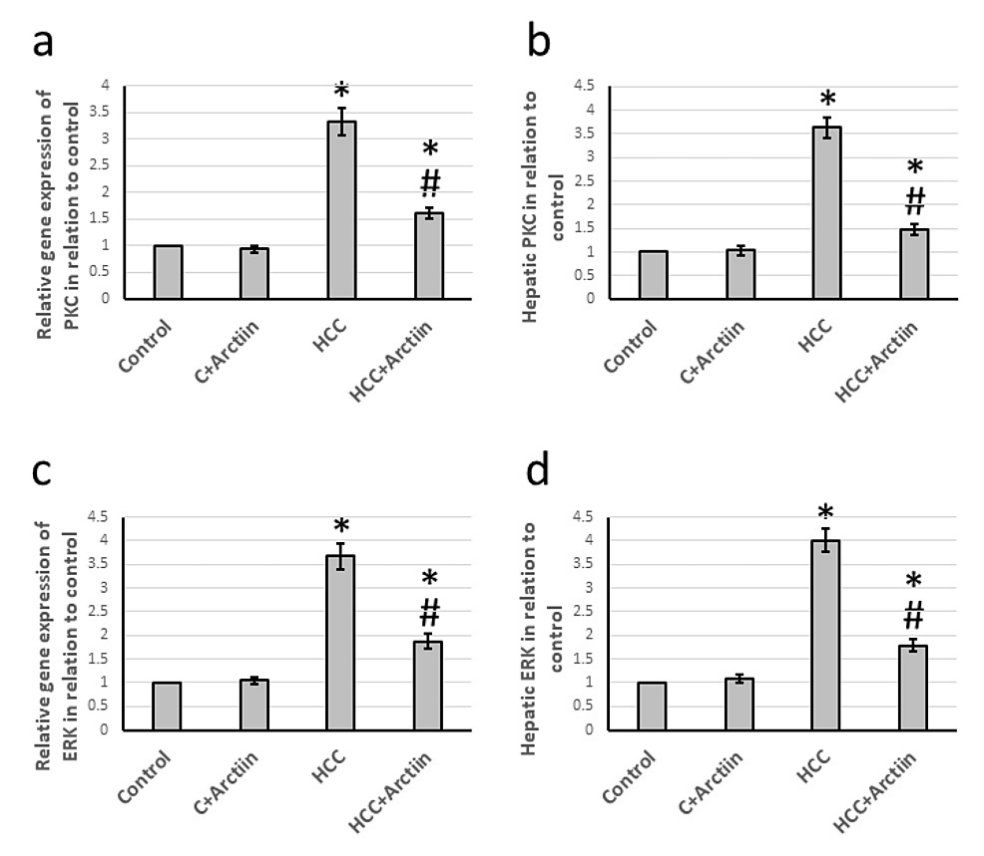
Effect of HCC and 30 mg/kg arctiin on hepatic gene expression of PKC (a) and ERK (c), as well as protein expression of PKC (b) and ERK (d). *Significant difference as compared with the control group at p<0.05. #Significant difference as compared with the HCC group at p<0.05 C, control; ERK, Extracellular signal-regulated kinase; HCC, hepatocellular carcinoma; PKC, protein kinase C

After studying hepatic tissues from rats with HCC, it was discovered that the gene expression of PKC and ERK increased by 3.33 and 3.67 times, respectively. This increase led to a rise in hepatic tissue levels of PKC and ERK by 3.63 and 4.01 times, respectively, as compared to the control group. However, arctiin was found to reverse all these effects in HCC rats while having no effect on the control rats (Figure 5).

## Discussion

Even though chemotherapy can be effective in treating HCC, it can also lead to severe side effects and is costly. In order to assess potential anti-tumor compounds, we instigated HCC in rats using thioacetamide. The findings indicated a significant reduction in the survival rate, with only 20% of the rats surviving. Moreover, there was an increase in the average number of nodules in the liver and serum AFP levels. Additionally, microsections stained with Masson trichrome from the HCC group demonstrated an enlarged area of fibrosis.

Arctiin is a naturally occurring active constituent that has been found to possess several important pharmacological activities. Extensive research has indicated that arctiin has the potential to alleviate inflammation, chronic pain, and swelling in patients with osteoporosis [10]. Our study has focused on the effects of arctiin on HCC in rats. The results of our study have been remarkable, as we observed a significant increase in the survival rate of HCC rats treated with arctiin. Additionally, there was a significant reduction in the number of nodules and serum AFP levels in these rats. Furthermore, our observations of micro-sections from HCC rats treated with arctiin and stained with Masson's trichrome revealed a marked improvement in hepatic tissue, as well as a reduction in the fibrosis area. These findings suggest that arctiin may have a therapeutic potential in the treatment of HCC, and further studies are warranted to explore the mechanism of its antitumor activity.

HIF-1α is a protein that plays a crucial role in regulating gene expression in response to low oxygen levels. This protein has a short half-life and acts as the primary marker of hypoxia, which is a condition characterized by reduced oxygen supply to tissues. HIF-1 is composed of two subunits, HIF-1α and HIF-1β, which are present in almost all mammalian cells. However, only HIF-1α is strictly regulated by hypoxia and activated during hypoxic conditions [17]. When hypoxia occurs, the HIF-1α protein is stabilized and moves into the nucleus, where it binds to specific DNA sequences called hypoxia-responsive elements (HREs). This binding activates the transcription of genes that help cells adapt to low oxygen conditions, including those involved in angiogenesis, glucose metabolism, and apoptosis. In addition to its role in hypoxia, HIF-1α also plays a critical role in cancer progression by promoting tumorigenicity and angiogenesis [18]. Interestingly, HIF-1α protein is almost undetectable in normal cells under normoxic conditions, whereas it is highly activated in many tumors. This aberrant activation of HIF-1α in cancer cells is due to genetic mutations or epigenetic alterations that disrupt its normal regulation. As such, targeting HIF-1α signaling has emerged as a promising strategy for cancer therapy [19]. We found a significant increase in the gene expression and protein levels of HIF-1α in HCC rats associated with elevation in the area immuno-stained with anti-HIF-1α. All these effects were reversed by treating HCC rats with arctiin without affecting the control rats. However, no previous study illustrated the ability of arctiin to reduce the expression of HIF-1α in any disease.

The β-catenin protein is a complex molecule that plays a critical role in the development, growth, regeneration, and invasion of tumors. It facilitates cell-to-cell adhesion and activates extracellular matrix components, leading to the overexpression of downstream target genes and ultimately resulting in carcinogenesis [20]. One of the downstream proteins of β-catenin is SMAD4, which is overexpressed in various types of cancer. Interestingly, deleting the SMAD4 gene has been found to have protective effects against pancreatic cancer [21]. We found a significant elevation in the expression of both β-catenin and SMAD4 in HCC rats, which was reduced by treating HCC rats with arctiin without affecting the control rats. Arctiin was reported previously to downregulate β-catenin, leading to inhibition of the growth of colon cancer cells. The activity depends on the 4-O-glucoside moiety [22]. We did not find any previous study that illustrates the ability of arctiin to inhibit SMAD4 in any disease model. In addition, this is the first study that describes the ability of arctiin to inhibit the β-catenin/SMAD4 pathway in HCC.

PKC enzymes are involved in numerous cellular processes, including cell growth, differentiation, and apoptosis. They are crucial for the regulation of signaling pathways that control cell proliferation, migration, invasion, tumorigenesis, and metastasis. PKC enzymes have been the subject of intense research in the field of cancer therapeutics due to their involvement in cancer progression [23]. For example, PKC stimulates the Ras/Raf/MEK/ERK signaling pathway, which plays a crucial role in cancer cell survival and proliferation. This pathway is frequently dysregulated in cancer cells, and PKC inhibitors have been shown to reduce tumor growth in preclinical studies [24]. Therefore, PKC enzymes and their downstream signaling pathways are attractive targets for the development of novel cancer therapeutics. Recent studies have provided compelling evidence suggesting a crucial role of the ERK pathway in the process of epithelial-mesenchymal transition (EMT) in HCC. EMT is a biological phenomenon characterized by the change of epithelial cells into mesenchymal cells, which plays a vital role in HCC invasion and metastasis [25]. An active ERK pathway has been found to promote the aggressiveness of HCC by facilitating EMT, while inhibiting the ERK pathway can effectively suppress HCC aggressiveness [26]. These findings have significant implications for HCC treatment as they provide a potential therapeutic target for controlling the progression of this deadly disease. However, we found a significant elevation in the expression of PKC and ERK in HCC rats, which was reduced by treating HCC rats with arctiin without affecting the control rats. No previous study illustrated the ability of arctiin to reduce the expression of PKC and ERK in animal models.

Limitations of the study

**Figure 6:**
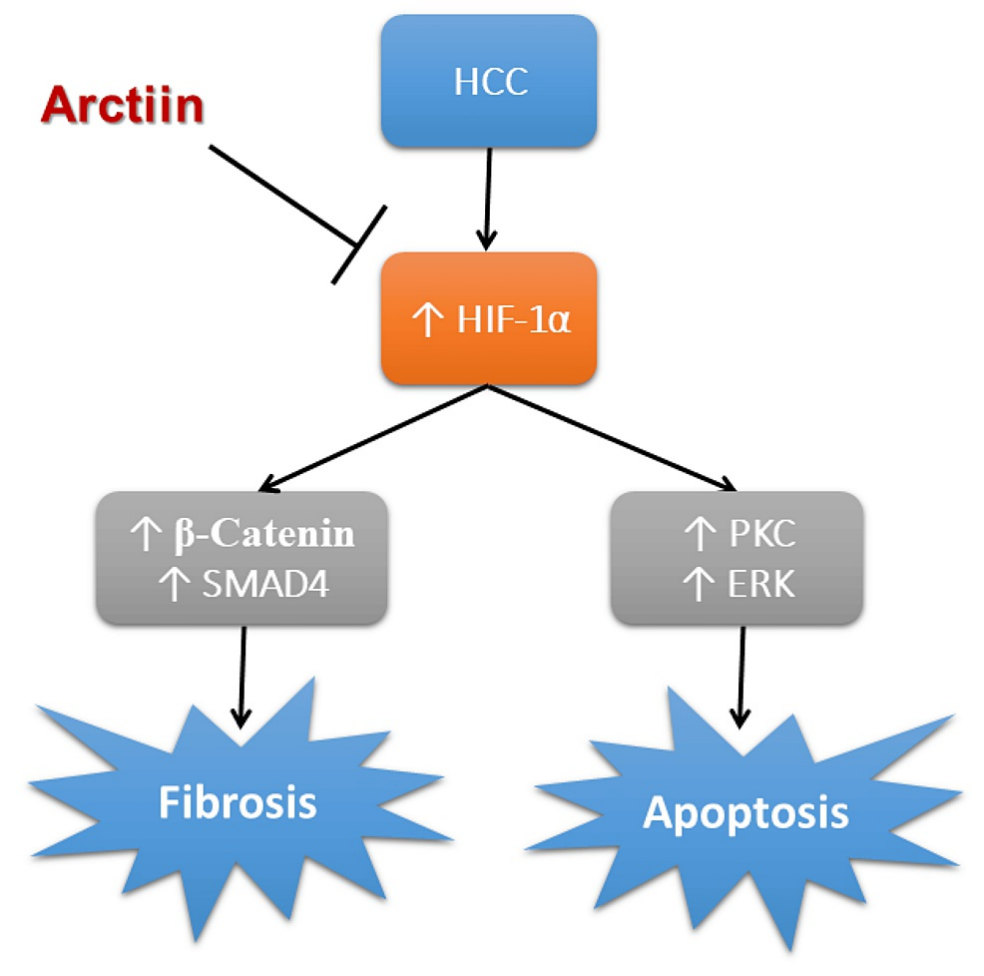
Schematic presentation of the protective effects of arctiin in HCC. ERK, extracellular signal-regulated kinase; HCC, hepatocellular carcinoma; HIF-1α, hypoxia-induced factor-1α; SMAD4, mothers against decapentaplegic homolog 4; PKC, protein kinase C The image was created by the authors of this study.

A summary of the antitumor effects of arctiin against HCC is provided in Figure 6. However, there are some limitations to the research due to the use of rats as a model organism. Rats have different metabolic pathways and drug metabolites than humans, which can lead to different dosing and various ways of the body dealing with the drugs. Therefore, the results obtained from this study should be interpreted with caution when extrapolating to humans. In addition to that, it is important to note that there are many methods for tumor induction in rats, but for this study, only the chemical induction of HCC in rats using thioacetamide was used. This means that the results may not be generalizable to other types of HCC induction methods. Despite these limitations, the study provides valuable insight into the potential antitumor effects of arctiin and could serve as a basis for future investigations in humans.

## Conclusions

Arctiin is a natural substance that has been found to possess anti-tumor properties that can increase survival rates, reduce the number of tumors, and decrease AFP levels in the serum. These benefits are attributed to its ability to block the expression of HIF-1α, a protein that plays a role in developing HCC-induced hypoxia. In addition, arctiin has been shown to slow down tumor fibrosis by decreasing the expression of two proteins, β-catenin and SMAD4. By doing so, the compound can help improve liver function and reduce the risk of liver failure. Moreover, arctiin has been found to reduce hepatic tissue apoptosis by downregulating two enzymes, PKC and ERK. This effect can help preserve liver health and prevent liver disease progression.
